# A distance based multisample test for high-dimensional compositional data with applications to the human microbiome

**DOI:** 10.1186/s12859-020-3530-x

**Published:** 2020-12-03

**Authors:** Qingyang Zhang, Thy Dao

**Affiliations:** Department of Mathematical Sciences, University of Arkansas, Fayetteville, AR 72701 USA

**Keywords:** Microbiome, Compositional data, High dimensionality, Centered log-ratio transformation, Multisample test, Distance correlation

## Abstract

**Background:**

Compositional data refer to the data that lie on a simplex, which are common in many scientific domains such as genomics, geology and economics. As the components in a composition must sum to one, traditional tests based on unconstrained data become inappropriate, and new statistical methods are needed to analyze this special type of data.

**Results:**

In this paper, we consider a general problem of testing for the compositional difference between K populations. Motivated by microbiome and metagenomics studies, where the data are often over-dispersed and high-dimensional, we formulate a well-posed hypothesis from a Bayesian point of view and suggest a nonparametric test based on inter-point distance to evaluate statistical significance. Unlike most existing tests for compositional data, our method does not rely on any data transformation, sparsity assumption or regularity conditions on the covariance matrix, but directly analyzes the compositions. Simulated data and two real data sets on the human microbiome are used to illustrate the promise of our method.

**Conclusions:**

Our simulation studies and real data applications demonstrate that the proposed test is more sensitive to the compositional difference than the mean-based method, especially when the data are over-dispersed or zero-inflated. The proposed test is easy to implement and computationally efficient, facilitating its application to large-scale datasets.

## Background

Data that lie on the simplex $\mathcal {S}^{d-1}=\big \{ (x_{1}, x_{2},..., x_{d}), s.t. \min _{j}x_{j}\geq 0, \sum _{j=1}^{d}x_{j}=1 \big \}$ are often called (*d*−1)-dimensional compositional data, and they arise in many scientific disciplines such as genomics, geology and economics [1–3]. As the components in a composition must sum to one, classic statistical tests including two-sample t-test and Wilcoxon rank-sum test become inappropriate as they require unconstrained data, and directly applying these standard methods to compositional data could result in misleading inference [4]. To overcome this difficulty, Aitchison (1982) proposed to use a log-ratio transformation to relax the unit-sum constraint, so that some classic tests can be applied to the transformed data. For instance, the generalized likelihood ratio test based on log-ratios [4] has been widely used to test compositional difference between groups due to its simplicity and good empirical performance.

It is noteworthy that Aitchison’s test only applies to low-dimensional settings where the dimension is less than sample size. In recent microbiome and metagenomic studies, however, the compositional data are often high-dimensional. For instance, in the Human Microbiome Project, it is common to have hundreds to thousands of bacterial taxa while only tens of samples are available for analysis. To this end, Cao et al. (2017) developed a powerful two-sample test for high-dimensional means using a centered log-ratio transformation [3]. Cao et al.’s test achieves satisfactory statistical power under high-dimensional sparse settings, and the consistency of the test has been well established under some regularity conditions. Nevertheless, this test has several shortcomings which has limited its application in practice. For instance, Cao et al.’s test can only deal with two-sample comparison, and its validity depends on a list of regularity conditions on the underlying covariance matrices. In addition, this test is a maximum-type test, and its performance relies on the sparsity assumption, i.e., only a small proportion of components in the composition are different across groups.

In this paper, we formulated a new hypothesis from a Bayesian point of view, to handle high-dimensionality and over-dispersion that are commonly seen in recent microbiome data. Different from those mean-based hypotheses, we assumed that the abundances follow a multinomial model with random composition parameters, and redefined the compositional equivalency using the distribution of random compositions. To directly target the distributional difference in composition, we suggested a distance based nonparametric test for detecting the difference between multiple groups. Unlike most existing tests for compositional data, our method does not rely on any data transformation, sparsity assumption or regularity conditions on the covariance matrix, but directly analyzes the compositions. Simulation studies demonstrated that our test is more sensitive to the compositional difference than the mean-based method, especially when the data are over-dispersed or zero-inflated. The proposed test is easy to implement and computationally efficient, facilitating its application to large-scale datasets.

The remainder of the paper is structured as follows: “Methods” section formulates the hypothesis testing and introduces our distance based method. “Results” section compares the proposed method with a log-ratio based test using simulated data from negative binomial models. In addition, we apply the new method to two real datasets on human throat microbiome and intestinal microbiome. “Discussion” section discusses our method with some future perspectives. “Conclusions” section concludes the paper.

## Methods

### Problem formulation

In this part, we briefly reviewed the test by Cao et al. (2017), and then formulated our new hypothesis. We begin with the notations. Let *k*∈{1,2,...,*K*} be the group index and *j*∈{1,2,...,*p*} be the index of components in the composition. Denote by $\pmb {X}^{(k)}=\left (\pmb {X}^{(k)}_{1},..., \pmb {X}^{(k)}_{n_{k}}\right)^{T}$ the observed *n*_*k*_×*p* data matrix for group *k*, where $\pmb {X}^{(k)}_{i}=\left (X_{i1}^{(k)},..., X_{ip}^{(k)}\right)^{T}$ represents the composition for subject *i* that lie on the (*p*−1)-dimensional simplex. We assume that the observed compositional data *X*^(*k*)^ arise from a latent matrix $\pmb {W}^{(k)}=\left (\pmb {W}^{(k)}_{1},..., \pmb {W}^{(k)}_{n_{k}}\right)^{T} \left (\pmb {W}^{(k)}_{1},..., \pmb {W}^{(k)}_{n_{k}} \text {are iid samples}\right)$ by normalization

$$X_{ij}^{(k)}=\frac{W_{ij}^{(k)}}{\sum_{h=1}^{p}W_{ih}^{(k)}}, $$

where the unobserved *W*^(*k*)^ may refer to the true abundance of bacterial taxa for microbiome data.

As the true abundances *W*^(*k*)^ are generally unknown, Cao et al. (2017) formulated a testable hypothesis for two groups:

$$\begin{array}{*{20}l} H_{0}&: E\left(\log\left(\pmb{W}_{1}^{(1)}\right)\right)=E\left(\log\left(\pmb{W}_{1}^{(2)}\right)\right)+c\pmb{1}_{p}, \text{for~some~} c\in \mathbb{R},\\ H_{\alpha}&: E\left(\log\left(\pmb{W}_{1}^{(1)}\right)\right)\neq E\left(\log\left(\pmb{W}_{1}^{(2)}\right)\right)+c\pmb{1}_{p}, \text{for~any~} c\in \mathbb{R}, \end{array} $$

where 1_*p*_ stands for the vector of *p* ones. The hypothesis above is mean-based and it can be tested through a centered log-ratio transformation

$$Y_{ij}^{(k)}=\log\frac{X_{ij}^{(k)}}{\left(\Pi_{h=1}^{p}X_{ih}^{(k)}\right)^{1/p}}, k=1,2; i=1,...,n_{k}. $$

It can be shown that the centered log-ratio variables $Y_{ij}^{(k)}$’s are only weakly dependent and satisfy certain desired properties, and Cao et al. (2017) suggested the following test statistics based on these log-ratio variables

$$\mathcal{M}_{n}=\frac{n_{1}n_{2}}{n_{1}+n_{2}}\max_{1\leq j\leq p}\frac{\left(\bar{Y}_{j}^{(1)}-\bar{Y}_{j}^{(2)}\right)^{2}}{\hat{\gamma}_{jj}}, $$

where $\bar {Y}_{j}^{(k)}=\sum _{i=1}^{n_{k}}Y_{ij}^{(k)}/n_{k}, \hat {\gamma }_{jj}=\sum _{k=1}^{2}\sum _{i=1}^{n_{k}}\left (Y^{(k)}_{ij}-\bar {Y}_{j}^{(k)}\right)^{2}/(n_{1}+n_{2})$. The *p*-value can be then obtained through Gumbel distribution

$$p=1-\left\{\exp\exp\left(-\frac{1}{2}\mathcal{M}_{n}-2\log p+\log\log p+\log\pi\right)\right\}^{-1}. $$

Cao et al.’s test targets the difference in high-dimensional means, and its validity relies on several assumptions on the underlying covariance matrices, which are impractical to check in reality. Here, we considered a different hypothesis based on the distribution of composition instead of means. Under multinomial model, we have

$$\pmb{W}^{(k)}_{i}\sim \text{Multinomial}\left(N^{(k)}_{i}, ~\pmb{\pi}^{(k)}_{i}\right), $$

where $N^{(k)}_{i}$ stands for the total abundance of bacterial taxa for sample *i* from group *k*, and $\pmb {\pi }^{(k)}_{i}$ represents the true composition. In order to model over-dispersion, we assumed random parameters, $N^{(k)}_{i}\sim f_{N}(\alpha)$ and $\pmb {\pi }^{(k)}_{i}=\left (\pi ^{(k)}_{i1},..., \pi ^{(k)}_{ip})\sim f_{\pi }(\pmb {\Theta }^{(k)}\right)$, where *α* and *Θ*^(*k*)^ are hyper-parameters. We then define the compositional equivalence between two groups based on the distribution of parameter *π*:

#### **Definition 1**

Two groups *k* and *k*^′^ are said to be compositionally equivalent if $\phantom {\dot {i}\!}f_{\pi }\left (\pmb {\Theta }^{(k)}\right)=f_{\pi }\left (\pmb {\Theta }^{(k')}\right)$.

By Definition 1, we formulate the null and alternative hypotheses for *K* groups

$$\begin{array}{*{20}l} H_{0}&: f_{\pi}\left(\pmb{\Theta}^{(1)}\right)=...=f_{\pi}\left(\pmb{\Theta}^{(K)}\right),\\ H_{\alpha}&: f_{\pi}\left(\pmb{\Theta}^{(k)}\right)\neq f_{\pi}\left(\pmb{\Theta}^{(k')}\right)~\text{for~some}~k~\text{and}~k'. \end{array} $$

Throughout this paper, we assume that the total abundance or sequencing depth $N^{(k)}_{i}$ is independent of $\pmb {\pi }^{(k)}_{i}$, and $N^{(k)}_{i}\sim f_{N}(\alpha)$ for *i*∈{1,...,*n*_*k*_} and *k*∈{1,2,...,*K*}, therefore testing *H*_0_ amounts to testing the distributional equality of the compositions between *K* groups. Let $f^{(k)}_{\pmb {X}}(\pmb {x})$ be the density function of $\pmb {X}^{(k)}_{i}$, one can test the following equivalent hypothesis

$$\begin{array}{*{20}l} H^{*}_{0}&: f^{(1)}_{\pmb{X}}(\pmb{x})=...=f^{(K)}_{\pmb{X}}(\pmb{x})~\text{for~all}~\pmb{x},,\\ H^{*}_{\alpha}&: f^{(k)}_{\pmb{X}}(\pmb{x})\neq f^{(k')}_{\pmb{X}}(\pmb{x})~\text{for~some}~\pmb{x},~k~\text{and}~k', \end{array} $$

Here, it is noteworthy that $H^{*}_{0}$ is equivalent to the independence between the composition *X* and the grouping variable *k*∈{1,2,...,*K*} (i.e., phenotype), which converts the problem to testing the independence between the continuous random vector and a categorical variable.

### Distance based test

In this part, we proposed a distance based method to test $H^{*}_{0}$, i.e., to detect the association between composition and phenotype. To begin with, we briefly introduce the notion of distance covariance. The distance covariance between two random vectors *X* and *Y* (can be of different sizes and different types) is defined as the square root of

1 $$ \text{dCov}^{2}(\pmb{X}, \pmb{Y}) = \int_{R^{d_{x}+d_{y}}}\frac{\|\phi_{\pmb{x}, \pmb{y}}(\pmb{t}, \pmb{s})-\phi_{\pmb{x}}(\pmb{t})\phi_{\pmb{y}}(\pmb{s})\|^{2}}{c_{d_{x}}c_{d_{y}}\|\pmb{t}\|^{1+d_{x}}_{d_{x}}\|\pmb{s}\|^{1+d_{y}}_{d_{y}}}d\pmb{t}d\pmb{s},  $$

where *ϕ*(·) represents a characteristic function, *d*_*x*_ and *d*_*y*_ are the dimensions of *X* and *Y*, $c_{d_{x}}=\frac {\pi ^{(1+d_{x})/2}}{\Gamma \{(1+d_{x})/2\}}$ and $c_{d_{y}}=\frac {\pi ^{(1+d_{y})/2}}{\Gamma \{(1+d_{y})/2\}}$. Unless otherwise specified, $\|\pmb {z}\|_{d_{z}}$ denotes the Euclidean norm of $\pmb {z}\in \mathbb {R}^{d_{z}}$, and $\|\phi \|^{2} = \phi \bar {\phi }$ for a complex-valued function *ϕ* and its conjugate $\bar {\phi }$.

One remarkable property of distance covariance is that dCov(X, Y)=0 if and only if *X* and *Y* are statistically independent, indicating that the distance covariance can also capture nonlinear associations. In their seminal work, Szekely et al. (2007) also provided the following alternative definition of distance covariance based on Euclidean distance and established its equivalency to the original definition in Eq. (1) (see Theorem 1, [5]):

$$\text{dCov}^{2}(\pmb{X}, \pmb{Y})= \text{Cov}(\|\pmb{X}_{1}-\pmb{X}_{2}\|, \|\pmb{Y}_{1}-\pmb{Y}_{2}\|)-2\text{Cov}(\|\pmb{X}_{1}-\pmb{X}_{2}\|, \|\pmb{Y}_{1}-\pmb{Y}_{3}\|), $$

where (*X*_1_,*Y*_1_), (*X*_2_,*Y*_2_) and (*X*_3_,*Y*_3_) be three independent copies of (*X*,*Y*). Here, we choose to use this alternative definition to derive the explicit formula of distance covariance between composition and phenotype. For ease of notations, let *Y* be the phenotype, taking values from a discrete set {1,2,...,*K*} with probabilities {*p*_1_,...,*p*_*K*_}, and *X*={*X*_1_,...,*X*_*p*_} be the composition. For illustration purpose, here we assume *Y* is nominal (without ordering between categories), however, our test can be easily extended to ordinal *Y* and the formula is given in the “Discussion” section. Let (*X*_1_,*Y*_1_), (*X*_2_,*Y*_2_) and (*X*_3_,*Y*_3_) be three independent copies of (*X*,*Y*), we define ∥*Y*_1_−*Y*_2_∥=1, if *Y*_1_≠*Y*_2_ and 0 otherwise. In addition, we define expected inter-point distance as

$$\mathcal{D}_{ij}=E(\|\pmb{X}_{1}-\pmb{X}_{2}\||Y_{1}=i, Y_{2}=j), i, j=1,...,K.$$

The distance covariance between *Y* and *X* can then be derived from the second definition

$$\begin{array}{*{20}l} E(\|Y_{1}-Y_{2}\|)&=1-\sum_{i=1}^{K}p^{2}_{i},\\ E(\|\pmb{X}_{1}-\pmb{X}_{2}\|)&=\sum_{i=1}^{K}\sum_{j=1}^{K}p_{i}p_{j}\mathcal{D}_{ij},\\ E(\|\pmb{X}_{1}-\pmb{X}_{2}\|\|Y_{1}-Y_{2}\|)&=\sum_{i\neq j}p_{i}p_{j}\mathcal{D}_{ij}=\sum_{i=1}^{K}\sum_{j=1}^{K}p_{i}p_{j}\mathcal{D}_{ij}-\sum_{i=1}^{K}p^{2}_{i}\mathcal{D}_{ii},\\ E(\|\pmb{X}_{1}-\pmb{X}_{2}\|\|Y_{1}-Y_{3}\|)&=\sum_{j=1}^{K}\sum_{i\neq l}p_{i}p_{j}p_{l}\mathcal{D}_{ij}=\sum_{i=1}^{K}\sum_{j=1}^{K}p_{i}(1-p_{i})p_{j}\mathcal{D}_{ij}. \end{array} $$

Summarizing the results above, we have

$$\text{dCov}(\pmb{X}, Y)=2\sum_{i=1}^{K}\sum_{j=1}^{K}p_{i}^{2}p_{j}\mathcal{D}_{ij}-\sum_{i=1}^{K}p^{2}_{i}\mathcal{D}_{ii}-\left(\sum_{i=1}^{K}p^{2}_{i}\right)\left(\sum_{i=1}^{K}\sum_{j=1}^{K}p_{i}p_{j}\mathcal{D}_{ij}\right). $$

By Cauchy-Schwarz inequality, it can be shown that dCov(*X*,*Y*)≥0 and the equality holds if and only if $\mathcal {D}_{ii}=\mathcal {D}_{jj}=\mathcal {D}_{ij}$ for all (*i*,*j*)’s. When *K*=2, we have the following special case

$$\text{dCov}(\pmb{X}, Y)=2p^{2}(1-p)^{2}(2\mathcal{D}_{12}-\mathcal{D}_{11}-\mathcal{D}_{22}).$$

The sample version of dCov(*X*,*Y*) can be expressed as

$$\widehat{\text{dCov}(\pmb{X}, Y)}=2\sum_{i=1}^{K}\sum_{j=1}^{K}\hat{p}_{i}^{2}\hat{p}_{j}\hat{\mathcal{D}}_{ij}-\sum_{i=1}^{K}\hat{p}^{2}_{i}\hat{\mathcal{D}}_{ii}-\left(\sum_{i=1}^{K}\hat{p}^{2}_{i}\right)\left(\sum_{i=1}^{K}\sum_{j=1}^{K}\hat{p}_{i}\hat{p}_{j}\hat{\mathcal{D}}_{ij}\right). $$

Let *n*_*i*_ be the sample size in group *i*, the maximum likelihood estimate of *p*_*i*_ is $\hat {p}_{i}=n_{i}/n$, and the sample inter-point distance can be computed as follows:

2 $$\begin{array}{*{20}l} \hat{\mathcal{D}}_{ij}&=\frac{1}{n_{i}n_{j}}\sum_{m=1}^{n_{i}}\sum_{l=1}^{n_{j}}\|\pmb{X}^{(i)}_{m}-\pmb{X}^{(j)}_{l}\|, \end{array} $$

3 $$\begin{array}{*{20}l} \hat{\mathcal{D}}_{ii}&=\frac{2}{n_{i}(n_{i}-1)}\sum_{m=1}^{n_{i}}\sum_{l=1}^{n_{i}}\|\pmb{X}^{(i)}_{m}-\pmb{X}^{(i)}_{l}\|, \end{array} $$

where $\left \{\pmb {X}^{(i)}_{1},...,\pmb {X}^{(i)}_{n_{i}}\right \}$ and $\left \{\pmb {X}^{(j)}_{1},...,\pmb {X}^{(j)}_{n_{j}}\right \}$ stand for samples of *X*_*i*_ and *X*_*j*_, respectively.

As the distribution of sample distance covariance is impractical to evaluate [5], we suggest a simple permutation procedure to obtain *p*-values. In practice, one can randomly shuffle the vector of *Y* for *M* times, and calculate sample distance covariance between composition and the permuted *Y*, then the permutation *p*-value can be computed as the proportion of distance covariance from permuted data that exceed the observed one.

It is noteworthy that in addition to distance correlation, there are many other dependence measures that could be used in our framework, including the energy-divergence metric [6], multiscale graph correlation [7] and projection correlation [8], among others. One may refer to Josse and Holmes (2014) [9] for a general review of existing dependence measures between random vectors, and Szekely and Rizzo (2013) [10] for a review of energy- and distance-based measures.

## Results

### Numerical study

We conduct three simulation studies to compare the distance based test and the log-ratio based test [3] in detecting the compositional differences between groups. In the first study, we focus on two-group comparison under various high-dimensional and over-dispersed models. The dimension is fixed at *p*=200 and two different sample sizes *n*_1_=*n*_2_=50 and *n*_1_=*n*_2_=100 are used. The abundance $W_{ij}^{(k)}$ are generated from three different settings

- **Setting 1**: $W_{ij}^{(k)} \sim \text {NegBin}\left (\mu ^{(k)}_{j}, r^{(k)}_{j}\right)$, *i*=1,...,*n*_*i*_, *j*=1,...,*p*, $r^{(1)}_{j} \sim \text {Unif}(0.1, 1), r^{(2)}_{j}=r^{(1)}_{j}, \mu ^{(1)}_{j} \sim \text {Unif}(10, 15)$. Let *I*={*I*_+_,*I*_−_} be the set of taxa with different abundances in two conditions, $\mu ^{(2)}_{j}=\mu ^{(1)}_{j}+\Delta $ for *j*∈*I*_+_ and $\mu ^{(2)}_{j}=\mu ^{(1)}_{j}-\Delta $ for *j*∈*I*_−_, $\mu ^{(2)}_{j}=\mu ^{(1)}_{j}$ for *j*∉*I*, |*I*_+_|=|*I*_−_|=*dp*, where |·| represents set cardinality, and *d* is the proportion of differential means. We chose *d*=5*%*,20*%*, representing relatively sparse and dense signals in mean difference, and used *Δ*={0.5,1.0,1.5,2.0,2.5,3.0}.
- **Setting 2**: Same as Setting 1, but $\mu ^{(1)}_{j} \sim \text {Unif}(5, 10)$.
- **Setting 3** (Negative binomial model with excess zeros): $W_{ij}^{(k)}=0$ with probability *π* (*π*=10*%*,20*%*), and $W_{ij}^{(k)} \sim \text {NegBin}\left (\mu ^{(k)}_{j}, r^{(k)}_{j}\right)$ with probability 1−*π*. Other settings are same as in Setting 1, and we used *d*=10*%*, *Δ*={0.5,1.0,1.5,2.0,2.5} in the simulation.

We compute the composition $X_{ij}^{(k)}$ by normalizing the abundance $W_{ij}^{(k)}$, and test the null hypothesis using compositional data at the level of 0.05. For Cao et al.’s test, we calculate the test statistics $\mathcal {M}_{n}$ and directly compute the *p*-value using Gumbel distribution. For our distance correlation test, *p*-value is computed based on 5,000 permutations.

**Fig. 1:**
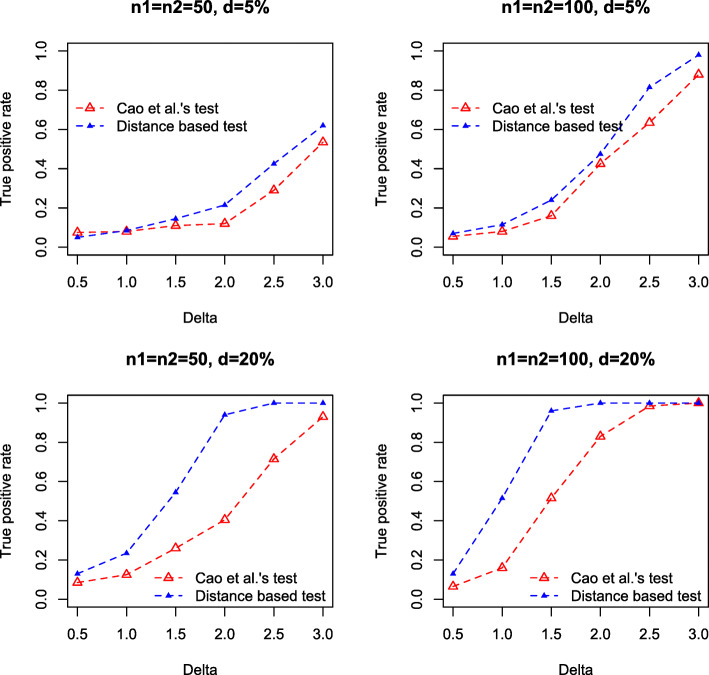
True positive rate against mean difference *Δ*, by Cao et al.’s test (red) and our test (blue) in Setting 1. Results are based on 1,000 simulations

**Fig. 2:**
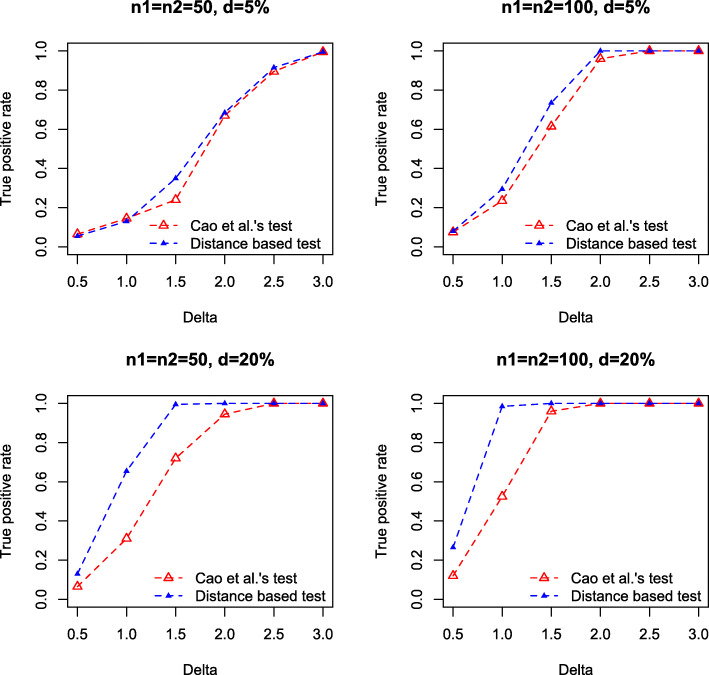
True positive rate against mean difference *Δ*, by Cao et al.’s test (red) and our test (blue) in Setting 2. Results are based on 1,000 simulations

**Fig. 3:**
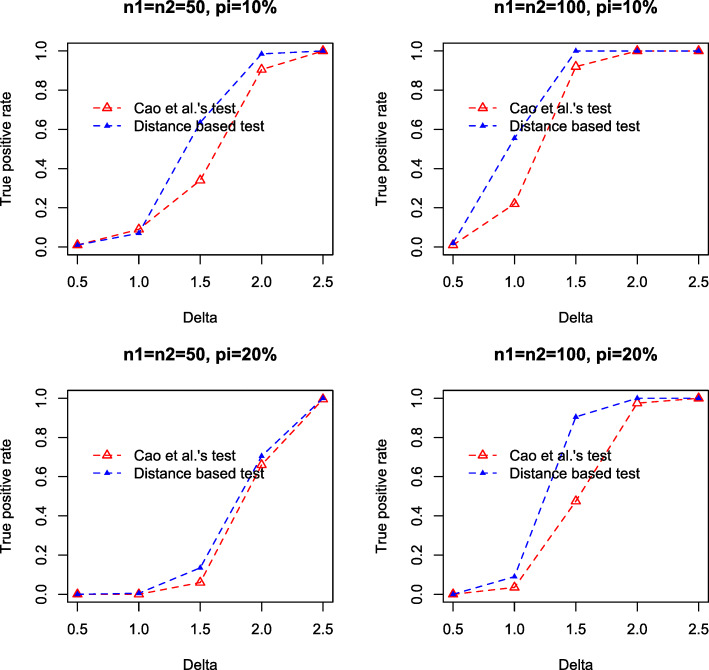
True positive rate against mean difference *Δ*, by Cao et al.’s test (red) and our test (blue) in Setting 3. Results are based on 1,000 simulations

For each setting, we simulate 1,000 datasets, and compare the true positive rates (TPRs) by the two tests. Figures 1, 2 and 3 summarize the TPRs under three settings. It can be seen that our distance based test consistently outperforms the log-ratio based method in all settings. Particularly, in the dense setting (*d*=20*%*), our test achieves substantially higher TPR than the log-ratio test. For instance, in Setting 1, when *Δ*=2.0, *n*_1_=*n*_2_=50, our test achieves a high TPR of 0.97 while the TPR by log-ratio test is only 0.41. However, when *Δ* is subtle, e.g., *Δ*=0.50, both tests fail to detect the difference, even for relatively large sample size, e.g., *n*_1_=*n*_2_=100.

In the second simulation study, we investigate the effect of dimension on the true positive rate. The sample size is fixed at *n*_1_=*n*_2_=100, and the dimension *p* is varied from 100 to 500. The abundance $W_{ij}^{(k)}$ are generated from two different settings (similar to settings 1 and 3)

- **Setting 4**: $W_{ij}^{(k)} \sim \text {NegBin}\left (\mu ^{(k)}_{j}, r^{(k)}_{j}\right)$, *i*=1,...,*n*_*i*_, *j*=1,...,*p*, $r^{(1)}_{j} \sim \text {Unif}(0.1, 1), r^{(2)}_{j}=r^{(1)}_{j}, \mu ^{(1)}_{j} \sim \text {Unif}(10, 15)$. Let *I*={*I*_+_,*I*_−_} be the set of taxa with different abundances in two conditions, $\mu ^{(2)}_{j}=\mu ^{(1)}_{j}+1.5$ for *j*∈*I*_+_ and $\mu ^{(2)}_{j}=\mu ^{(1)}_{j}-1.5$ for *j*∈*I*_−_, $\mu ^{(2)}_{j}=\mu ^{(1)}_{j}$ for *j*∉*I*, |*I*_+_|=|*I*_−_|=10, where |·| represents set cardinality.
- **Setting 5** (Negative binomial model with excess zeros): $W_{ij}^{(k)}=0$ with probability *π*=10*%*, and $W_{ij}^{(k)} \sim \text {NegBin}\left (\mu ^{(k)}_{j}, r^{(k)}_{j}\right)$ with probability 1−*π*. Other settings are same as in Setting 4.

**Fig. 4:**
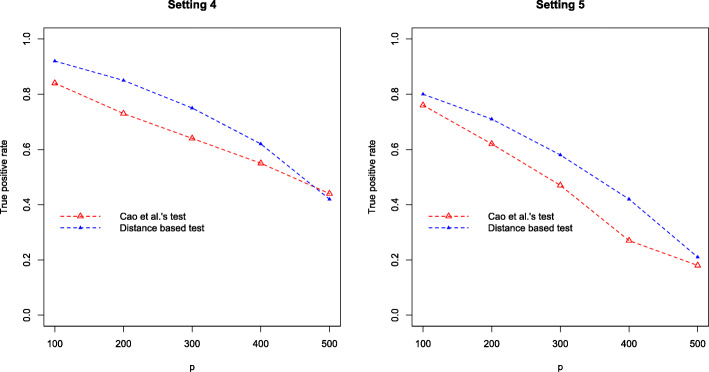
True positive rate against dimension *p*, by Cao et al.’s test (red) and our test (blue) in the second simulation study (settings 4 and 5). Results are based on 1,000 simulations

Figure 4 summarizes the TPRs by the two tests based on 1,000 replicates and significance level *α*=0.05. It can be seen that the distance based test outperforms the log-ratio test especially when the dimension is relatively low. When the dimension is high, for instance *p*=500, the two tests are comparable. More importantly, there is a substantial decrease of TPR as *p* increases, indicating that a feature screening could improve the test performance when *p* is large.

In the third study, we consider testing the compositional difference between multiple groups. We set *K*=4 with sample sizes *n*_1_=*n*_2_=*n*_3_=*n*_4_=50. The dimension *p* is fixed at 200. The abundance $W_{ij}^{(k)}$ are generated from the negative binomial model with excess zeros. Let $\pi =P\left (W_{ij}^{(k)}=0\right)$, with probability 1−*π*, $W_{ij}^{(k)} \sim \text {NegBin}\left (\mu ^{(k)}_{j}, r^{(k)}_{j}\right)$, *i*=1,...,*n*_*i*_, *j*=1,...,*p*, $r^{(1)}_{j} \sim \text {Unif}(0.1, 1), r^{(3)}_{j} \sim \text {Unif}(0.1, 1), r^{(2)}_{j}=r^{(1)}_{j}, r^{(4)}_{j}=r^{(3)}_{j}, \mu ^{(1)}_{j} \sim \text {Unif}(10, 15), \mu ^{(3)}_{j} \sim \text {Unif}(10, 15)$. Let *I*={*I*_+_,*I*_−_} be the set of taxa with different abundances in four conditions, $\mu ^{(2)}_{j}=\mu ^{(1)}_{j}+\Delta $ and $\mu ^{(4)}_{j}=\mu ^{(3)}_{j}+\Delta $ for *j*∈*I*_+_, $\mu ^{(2)}_{j}=\mu ^{(1)}_{j}-\Delta $ and $\mu ^{(4)}_{j}=\mu ^{(3)}_{j}-\Delta $ for *j*∈*I*_−_, $\mu ^{(1)}_{j}=\mu ^{(2)}_{j}=\mu ^{(3)}_{j}=\mu ^{(4)}_{j}$ for *j*∉*I*, |*I*_+_|=|*I*_−_|=20, where |·| represents set cardinality. We use *Δ*={0.5,1.0,1.5,2.0,2.5} and *π*={10*%*,20*%*} in the simulation.

**Fig. 5:**
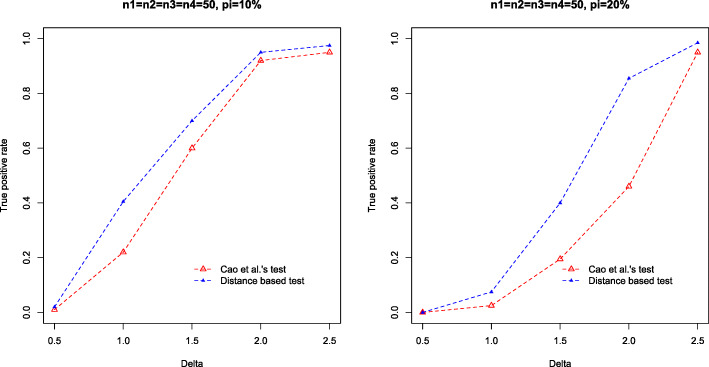
True positive rate against mean difference *Δ*, by Cao et al.’s test (red) and our test (blue) in the third simulation study (zero-inflated negative binomial and multi-sample comparison). Results are based on 1,000 simulations

For the distance correlation test, *p*-value is computed based on 5,000 permutations. For Cao et al.’s test, we calculate the *p*-values by Gumbel distribution from six pairwise comparisons, and use the smallest *p*-value for decision-making. Figure 5 summarizes the TPRs by the two tests, where it can be seen that our proposed test performs consistently better than the log-ratio based test. Notably, in the setting *π*=20*%* and *Δ*=2.0, the distance correlation test achieves a TPR of 0.83, compared to the TPR of 0.46 by the log-ratio test.

### Two microbiome applications

#### Analysis of throat microbiome data

In this part, we use the proposed hypothesis and distance based test to reanalyze a throat microbiome dataset. Cigarette smokers have an increased risk of infectious diseases involving the respiratory tract, however, the consequences for global airway microbial community composition remains unclear. Charlson et al. (2010) used culture-independent high-density sequencing to analyze the microbiota from the right and left nasopharynx and oropharynx of 29 smoking and 33 nonsmoking healthy adults to assess microbial composition and effects of cigarette smoking [11]. Bacterial communities were profiled using 454 pyrosequencing of 16S sequence tags, aligned to 16S rRNA databases.

We are interested in whether there is any significant difference in microbial compositions between smokers and non-smokers. The processed data (observed abundance) were downloaded from R package *GUniFrac* [12], which included the read counts of 856 predefined operational taxonomic units (OTUs, also called phylotypes) on 62 samples. We first deleted OTUs with extremely small number of reads (less than 20 reads in total), resulting a final set of 190 OTUs.

Two methods, including the log-ratio based test and distance based test, are applied to the compositional data. The proposed distance correlation test is implemented in the following steps

- Step 1: Compute the composition *X* for each sample by normalizing the abundance *W*.
- Step 2: Calculate the sample proportions $\hat {p_{i}}$, and the inter-group distances $\hat {\mathcal {D}}_{ij}$ and $\hat {\mathcal {D}}_{ii}$, *i*,*j*=1,...,*K* (e.g., Euclidean distance) using Eqs. (2) and (3).
- Step 3: Compute the permutation *p*-value based on $\widehat {\text {dCov}(\pmb {X}, Y)}$.

The proposed test yields a *p*-value of 0.0027, indicating a significant difference between smokers and non-smokers in microbial composition. In contrast, the test based on log-ratio transformation gives a *p*-value of 0.098, thus fails to reject the null hypothesis of equal means at the level of 0.05.

**Fig. 6:**
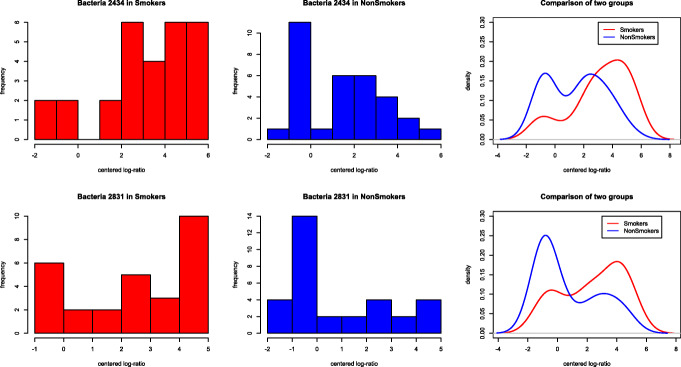
Histograms and fitted density curves of centered log-ratios of two OTUs (bacteria 2434 and bacteria 2831), red for smokers and blue for non-smokers

The disagreement between the two tests may indicate the existence of nonlinear effects and over-dispersion, because the log-ratio test only targets the mean difference while our test targets the distributional difference. We illustrate this point by carrying out additional analyses. Figure 6 gives two examples (bacteria 2434 and bacteria 2831), where the centered log-ratios exhibite substantially different distributions. However, the mean difference is not significant due to the nonlinear effect and heavy tails, which inflates the variance estimates in Cao et al.’s test.

**Fig. 7:**
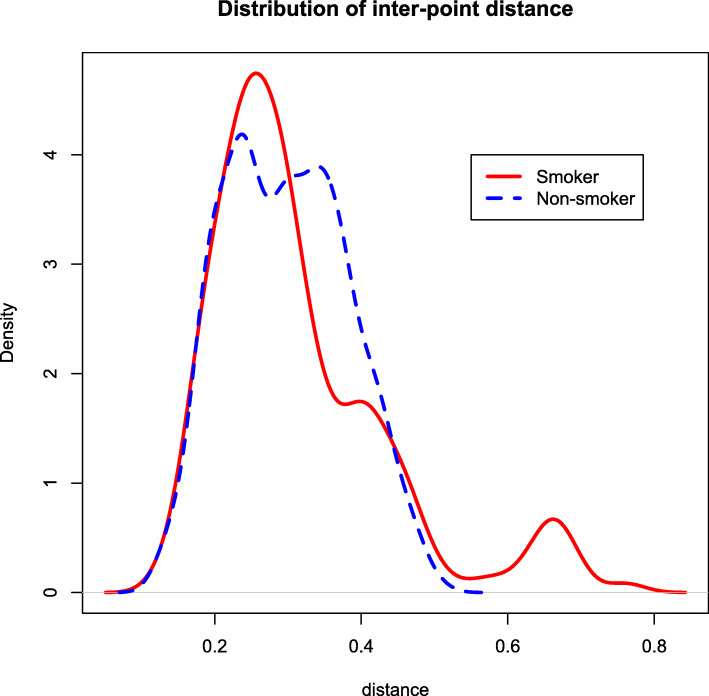
Distributions of inter-point distance within the smoking group (red solid) and non-smoking group (blue dashed)

**Fig. 8:**
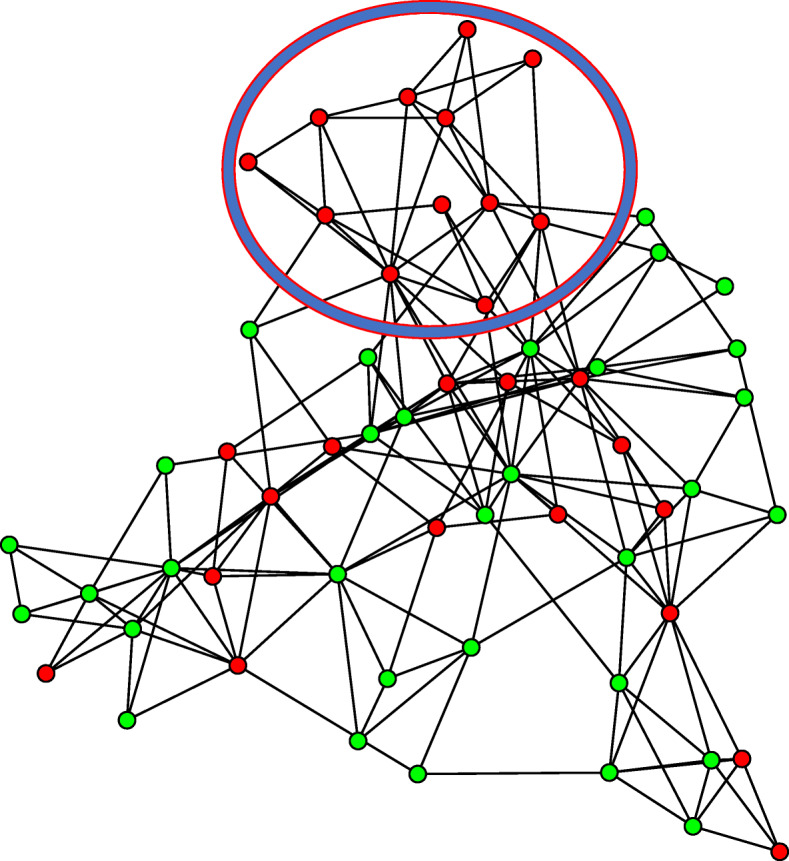
The 3-MST for all samples based on Euclidean distance, red nodes are for smokers and green nodes are for non-smokers. A connection between two samples represents compositional similarity

We also compare the distributions of inter-point distance within smokers and non-smokers. Szekely et al. (2007) illustrated that if two multivariate distributions are identical, the inter-point distances within each group have the same distribution. Figure 7 showed the inter-point distance distributions of two groups, where a substantial discrepancy was observed. Furthermore, we used the 3-minimum spanning tree (3-MST), a tree-based visualization method, to confirm our findings. Figure 8 shows the 3-MST based on the compositional data, where a connection in the network represents compositional similarity between two samples. In theory, if the two groups have the same distribution, then each sample has equal chance to connect with any other sample, regardless of which group it is from. However, it can be seen that certain samples from the same group formed clusters in the network. For instance, we identified a set of 12 smokers (circled) that are highly connected each other, but with very few connections with non-smokers, indicating a distributional difference in composition between the two groups.

#### Analysis of intestinal microbiome data

The microbial communities living in the human intestine have profound impact on our well-being and health. To understand the mechanisms that control this complex ecosystem, Lahti et al. (2014) conducted a deep phylogenetic analysis of the intestinal microbiota in 1,006 western adults from Europe and the United States [13]. The analysis is based on 130 genus-like phylogenetic groups that cover the majority of the known bacterial diversity of the human intestine. Clinical variables include age, nationality, BMI and DNA extraction method etc.

**Fig. 9:**
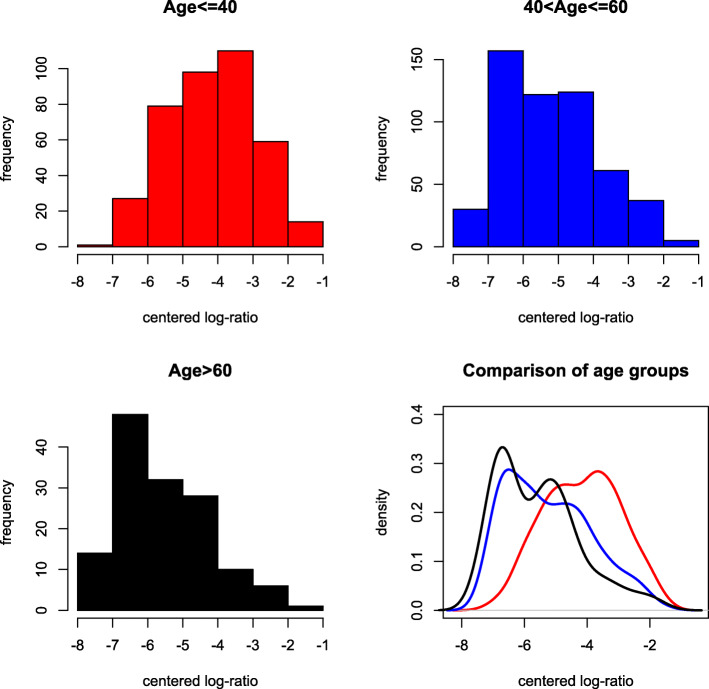
Histograms and fitted density curves of the log-ratios of group 25, red for young, blue for middle-aged and black for older subjects

**Fig. 10:**
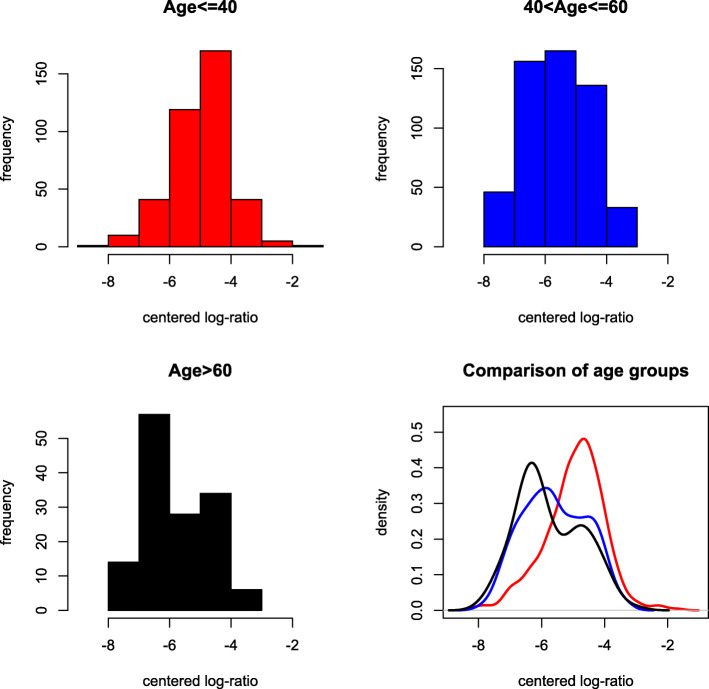
Histograms and fitted density curves of the log-ratios of group 60, red for young, blue for middle-aged and black for older subjects

**Fig. 11:**
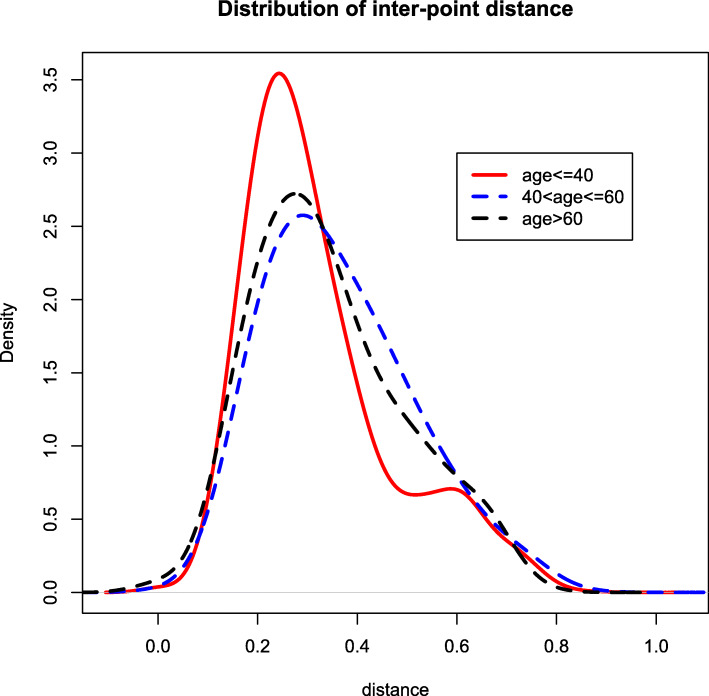
Distributions of inter-point distance within each age group (red solid for young, blue for middle-aged and black for older subjects)

One of the key research questions is whether different age groups have different microbiome compositions. We use the cutoffs suggested by Lahti et al. (2014) to define three age groups: young (18–40), middle-aged (41–60) and older (61–77). The distance test yields an overall *p*-value of 3.0×10^−6^. In addition, we calculate the *p*-values for the three pairwise comparisons: 8.2×10^−5^ for young vs middle aged, 2.2×10^−5^ for young vs older, and 0.081 for middle-aged vs older, indicating a significant difference in microbiome compositions between young and middle-aged/older subjects, but a minor difference between middle-aged and older subjects. To confirm this finding, we identified a list of microbiome groups with different distributions between age groups. Figures 9 and 10 show two examples of these, including group 25 and group 60. The distribution of inter-point distance within each age group is given in Fig. 11, where a discrepancy can be observed between young and middle-aged/older subjects.

## Discussion

Microbiome data are often compositional, high-dimensional and over-dispersed, which poses great challenges to the statistical analysis. To overcome these obstacles, in this work, we formulated a new testable hypothesis from a Bayesian point of view, and suggested a nonparametric test to detect the compositional difference between multiple populations. Compared to the existing tests, our method has several advantages. First, the distance based test is free of parametric assumptions but directly targets the distributional difference, therefore it is capable of detecting nonlinear effects. The application in throat microbiome provided a good example, where the new test successfully captured the difference between two phenotypes, while the mean based test failed to do so. In addition, our method can deal with multiple groups, while most of existing methods are only for two-group comparison. Third, our test does not require sparsity assumption on the mean differences as in Cao et al.’s test, and in our simulation study, the new test worked quite well against both sparse and relatively dense alternatives.

There are several possible extensions of the proposed test. First, the distance based method can be readily extended to ordinal phenotypes (or conditions), although we have been using nominal phenotypes for illustrative purpose. For ordinal phenotype, *Y*∈{1,2,...,*K*}, where there is a natural ordering 1<2...<*K*, (e.g., {mild, moderate, severe} for severity of a disease, {I, II, III, IV} for cancer stage, or {non-smoking, light smoking, heavy smoking} for smoking status), we need predefine the distance matrix between categories *i* and *j*, for instance, *d*_*ij*_=|*i*−*j*|, or *d*_*ij*_=|*i*−*j*|^2^. The distance covariance between composition *X* and ordinal phenotype *Y* has the following expression

$$\begin{aligned} \text{dCov}^{2}(\pmb{X}, Y)&=\left(\sum_{i=1}^{K}\sum_{j=1}^{K}p_{i}p_{j}d_{ij}\right)\left(\sum_{i=1}^{K}\sum_{j=1}^{K}p_{i}p_{j}D_{ij}\right)+\sum_{i=1}^{K}\sum_{j=1}^{K}p_{i}p_{j}d_{ij}D_{ij}\\&-2\sum_{i=1}^{K}\sum_{j=1}^{K}\sum_{l=1}^{K}p_{i}p_{j}p_{l}d_{il}D_{ij}, \end{aligned} $$

and one may use the same permutation procedure to obtain *p*-values. In practice, the distance matrix *d*_*ij*_ should be carefully chosen to reflect the true spacings between categories. An inappropriate choice of *d*_*ij*_ may result in misleading conclusions. Second, our test might be improved by incorporating more information about bacteria taxa. For instance, one can assign different weights for different bacterial taxa based on their position in the polygenetic tree [14], and use weighted Euclidean distance to construct the test statistic.

In addition to the microbiome application that we illustrated in this paper, the proposed test can be readily applied to several other fields. For instance, the market share data in economics are compositional and often high-dimensional [15]. One may apply our test to detect the market share difference between multiple countries. In geology, it is often of interest to study the compositions of species in sediment [16] and it is possible to apply our test to detect the difference in species compositions between multiple locations.

## Conclusions

We formulate a Bayesian testing framework to identify the compositional differences between multiple populations. In addition, we propose to use the distance correlation measure to test the null hypothesis. Simulation studies and two real applications in the human microbiome demonstrate that our test is more sensitive to the compositional difference than the mean-based method, especially when the data are over-dispersed or zero-inflated. The proposed test is easy to implement and computationally efficient, facilitating its application to large-scale datasets.

## Data Availability

The throat microbiome data can be downloaded from R package *GUniFrac* at https://cran.r-project.org/web/packages/GUniFrac/index.html. The intestinal microbiome data can be downloaded at https://datadryad.org/stash/dataset/doi:10.5061/dryad.pk75d.

## Abbreviations

dCov: Distance covariance
OTU: Operational taxonomic unit
MST: Minimum spanning tree
TPR: True positive rate
